# Unusually Large Extent of Pagetic Vertebral Ankylosis in a Patient with Ankylosing Spondylitis

**DOI:** 10.5334/jbsr.2542

**Published:** 2021-09-29

**Authors:** Iness Tirraf, Pierre-Antoine Poncelet

**Affiliations:** 1 Hôpital de Jolimont, BE; 2 Grand hôpital de Charleroi, BE

**Keywords:** Paget’s disease, ankylosing spondylitis, pagetic vertebral ankylosis

## Abstract

**Teaching point:** When the joints between bones are ankylosed, such as in ankylosing spondylitis, Paget’s disease can involve multiple contiguous bones leading to pagetic vertebral ankylosis.

## Case

A 76-year-old male was referred to our radiology department to perform an abdominal computed tomography (CT) as part of the follow-up after Whipple surgery for pancreatic intraductal papillary mucinous neoplasm. He had a prior history of Paget’s disease (PD) and ankylosing spondylitis (AS) and was totally asymptomatic.

**Figure 1:**
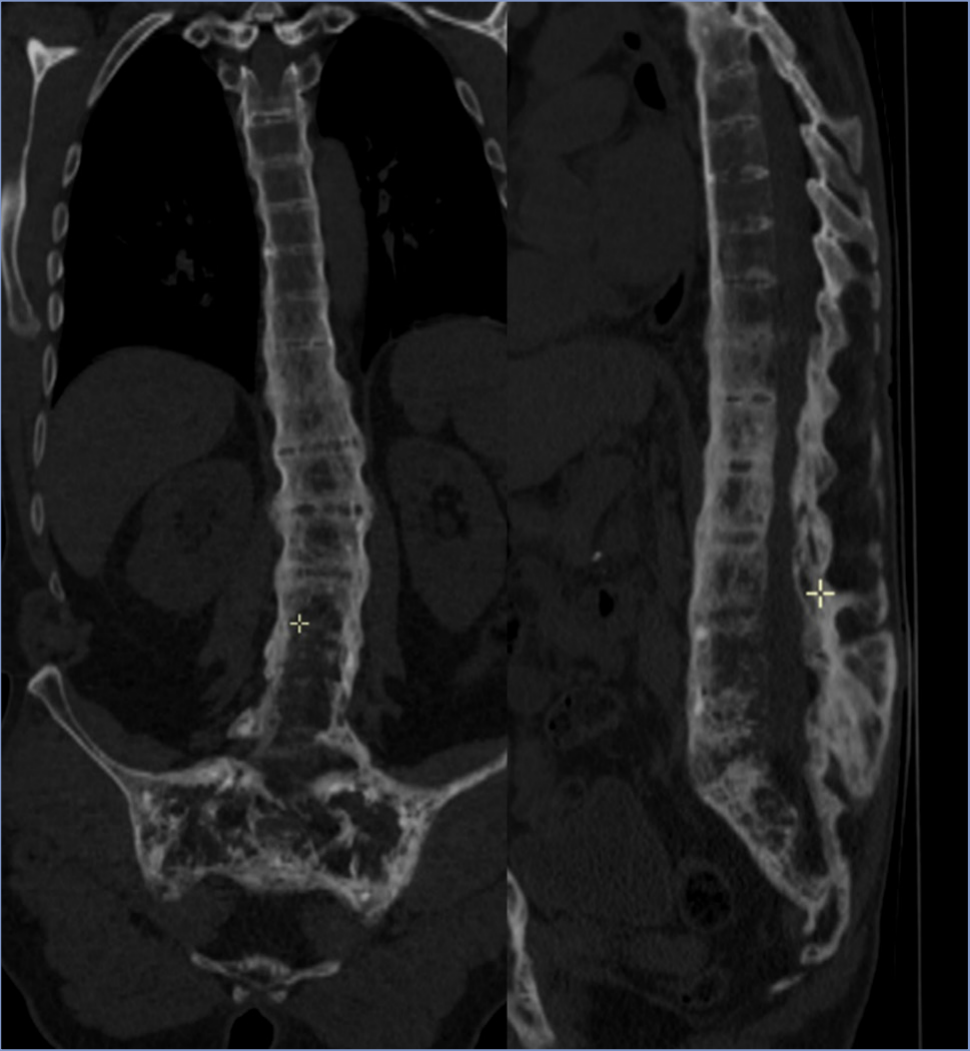


**Figure 2:**
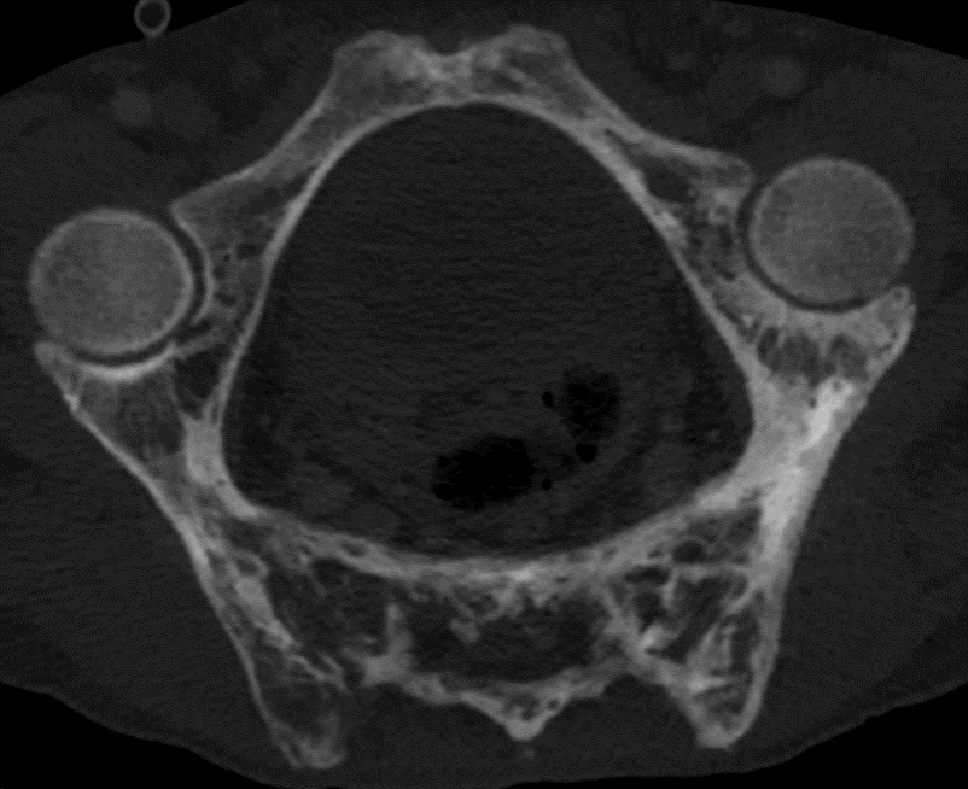


CT showed characteristic features of AS (syndesmophytes, vertebral squaring, fused costovertebral joints, and fused sacro-iliac joints) associated with typical bone remodeling features of PD (coarsening of the primary trabeculae, marginal vertebral sclerosis) resulting in an extensive pagetic vertebral ankylosis (PVA) with involvement of the dorsolumbar spine, sacrum (***Figure 1***), iliac bones (***Figure 2***), and costovertebral joints (not shown). MRI demonstrated a normal fatty bone marrow signal consistent with uncomplicated PD in the late or inactive state (not shown).

## Discussion

PD is a chronic condition characterized by constant bone remodeling involving osteoclastic and osteoblastic mechanisms. The most common sites of involvement are pelvis, lumbosacral spine, femur, or tibia and at least 50% of patients present with polyostotic disease [1].

Usually, PD does not progress to adjacent bones as the soft tissues act as natural barriers.

Some authors suggested a possible progression of the pagetic process through discs that presented signs of degeneration, but it remains unclear if intradiscal pagetic invasion is possible through healthy disc tissue. Otherwise, there is evidence of PVA in patients suffering from PD and a disease responsible of bone bridging such as AS or diffuse idiopathic skeletal hyperostosis. This case report suggests another hypothesis for the progression of the pagetic process to contiguous bones through syndesmophytes or osteophytes, leading to PVA [1].

In our case, there is a continuum of PD bone remodeling involving iliac bones, sacrum, thoraco-lumbar spine, and ribs. The extension through adjacent vertebral bodies is explained by the presence of syndesmophytes and the extension through iliac bones and ribs might be explained by the fusion of the sacro-iliac and costo-vertebral joints secondary to the AS inflammatory process.

Our findings support the hypothesis that PD may gradually progress in the contiguous ankylosed bones through acquired bone bridges and explain one of the most extensive PVA cases reported so far.
